# Quantitative Interpretation of Tracks for Determination of Body Mass

**DOI:** 10.1371/journal.pone.0077606

**Published:** 2013-10-30

**Authors:** Tom Schanz, Yvonne Lins, Hanna Viefhaus, Thomas Barciaga, Sashima Läbe, Holger Preuschoft, Ulrich Witzel, P. Martin Sander

**Affiliations:** 1 Lehrstuhl für Grundbau, Boden- und Felsmechanik, Ruhr-Universität Bochum, Bochum, Germany; 2 Steinmann-Institut für Geologie, Mineralogie und Paläontologie, Universität Bonn, Bonn, Germany; 3 Abteilung für Funktionelle Morphologie, Institut für Anatomie, Ruhr-Universität Bochum, Bochum, Germany; 4 Forschungsgruppe Biomechanik, Lehrstuhl für Maschinenelemente und Konstruktionslehre, Ruhr-Universität Bochum, Bochum, Germany; University of Pennsylvania, United States of America

## Abstract

To better understand the biology of extinct animals, experimentation with extant animals and innovative numerical approaches have grown in recent years. This research project uses principles of soil mechanics and a neoichnological field experiment with an African elephant to derive a novel concept for calculating the mass (i.e., the weight) of an animal from its footprints. We used the elephant's footprint geometry (i.e., vertical displacements, diameter) in combination with soil mechanical analyses (i.e., soil classification, soil parameter determination in the laboratory, Finite Element Analysis (FEA) and gait analysis) for the back analysis of the elephant's weight from a single footprint. In doing so we validated the first component of a methodology for calculating the weight of extinct dinosaurs. The field experiment was conducted under known boundary conditions at the Zoological Gardens Wuppertal with a female African elephant. The weight of the elephant was measured and the walking area was prepared with sediment in advance. Then the elephant was walked across the test area, leaving a trackway behind. Footprint geometry was obtained by laser scanning. To estimate the dynamic component involved in footprint formation, the velocity the foot reaches when touching the subsoil was determined by the Digital Image Correlation (DIC) technique. Soil parameters were identified by performing experiments on the soil in the laboratory. FEA was then used for the backcalculation of the elephant's weight. With this study, we demonstrate the adaptability of using footprint geometry in combination with theoretical considerations of loading of the subsoil during a walk and soil mechanical methods for prediction of trackmakers weight.

## Introduction

Since the first massive bones of sauropods were discovered, many scientists have investigated how these animals evolved to their gigantic size [1]–[3]. Analyses and interpretation of sauropod gigantism are essential for the understanding of evolutionary constraints and how these constraints impact Earth's geological and biological history. Bones of sauropods, of course, are not their only remains in the fossil record, but the second most common evidence for their former existence are footprints and entire trackways. The track record is important because it provides anatomical details and locomotion patterns of the trackmaker. Unlike bones, which are often transported, trace fossils are autochthonous and provide unequivocal information about the actual habitat of the trackmaker. The enormous tracks of gigantic sauropod dinosaurs occur in sediments from the Late Triassic [4] to Cretaceous all over the world [5]: e.g., in tidal flat deposits of the Paluxy River tracksite in Texas, USA [6]; in fluvial deposits [7], [8] and in lacustrine carbonate sediments of the Morrison Formation [9], [10] or in lagoonal deposits in Münchehagen, Germany [11], [12]. A comprehensive listing and review is found in [13].

In the past, mostly descriptive studies of tracks were done, but currently the focus is on understanding the paleobiology of the trackmaker. In general, it is possible to estimate anatomical details like hip heights [14] of the trackmaker from the tracks or to estimate walking velocity from measurements of pace and stride [15]–[17]. Modern vertebrate ichnology deals with experiments on living animals e.g., [18], [19], artificial indenters in the laboratory e.g., [20], [21], and computer-aided approaches e.g., [22], [23]. Common methods for calculating body mass based on body volume and density were done with models [24], 3D scanning [25], [26], or numerical methods [27]. Current numerical studies [28]–[31] have as their main objective to qualitatively better understand the kinematics of the foot indenting the subsoil and to relate subsoil properties to footprint quality and preservation.

Quantitative approaches to dinosaur footprints offer the perspective of addressing a fundamental question in dinosaur paleobiology, i.e., mass estimation. However, a reliable quantitative method for weight reconstruction from dinosaur footprints has not been developed so far, even though this is of major importance, especially for gigantic sauropods [32].

Here we introduce an approach for weight estimation based on footprint geometry using soil mechanical concepts. These can be used to back calculate the load applied to the subsoil by the trackmaker's feet. The geometry of the footprint (i.e., vertical displacements and diameter) is strongly influenced by the applied stress and the constitutive characteristics of the subsoil. Note that we use the term "geometry" in a different way than in the literature on dinosaur ichnology where it refers to the parameters of entire trackways. However, we only study the individual footprint, not the trackway. The value of the stress applied to the subsoil depends on the weight of the dinosaur (i.e., a static component) as well as on the deceleration that the dinosaur foot experiences when coming into contact with the subsoil (i.e., a dynamic component). In addition, biomechanical aspects, such as gait and weight distribution among the four limbs of the trackmaker, have to be taken into account when dealing with this problem. An important step towards the application of the soil-mechanical approach to fossil footprints is the validation by work on extant tracks, also known as the actualistic approach in paleontology. The African elephant (*Loxodonta africana*) is the largest terrestrial animal today, just as the sauropods were in the Mesozoic. Considering elephants and sauropods show similarities in foot morphology, quadrupedality and massive, graviportal limbs, elephants have often been included as recent analogs in sauropod research e.g., [19], [23]. The field part of our study was conducted at the Zoological Gardens Wuppertal, Germany. Briefly, after weighing an African elephant cow was walked across a prepared sand bed to produce footprints. Based on the footprint geometry, gait analysis and soil mechanical properties of the subsoil, the Finite Element Analysis (FEA) was adopted to back calculate the weight of the elephant. For simplicity, in this analysis we only consider layered subsoil properties that are homogenous within each layer. We are aware that the situation in track formation often is much more complex, especially for a foot penetrating soft layers in a large deformation type of kinematics before finding resistance at a competent layer below, see [30], [33]. For this study we focus on sand as subsoil material because in a next step we will target sauropod footprints preserved in sandstones.

Well known sauropod track sites in sandstones are the Late Jurassic sites of Barkhausen [34], [35] and Copper Ridge (Utah, USA) [7], [36], and the Early Cretaceous site of Münchehagen [11], [12], also Germany. Barkhausen shows several trackways of relatively small sauropods together with one theropod trackway in a fine-grained sand. The surface on which the animals walked is well preserved as indicated by the distinctive sediment bulges caused by the feet. The same applies to the Copper Ridge site which was made by a large sauropod that walked on a 15 cm thick bed of medium sand underlain by a mudstone. The Münchehagen site records numerous long trackways impressed in a 25 cm thick medium sandstone also underlain by a mudstone. Some of the tracks are partially eroded at this site, making them unsuitable for the soil mechanical approach to weight estimation. However, note that this paper only reports on a first step in methods development, showing that weight estimation from footprints is possible. Considerably more research is necessary before reliable results can be obtained for sauropods, let alone other dinosaurs. Note also that elephants and sauropods are particularly suitable for this approach because of their graviportal stance and locomotion and their simple foot morphology.

## Methods and Materials

For the present research, FEA, gait analysis and Digital Image Correlation (DIC) technique were carried out, the specifics of both of which are described below. The subsoil used in the field experiment was classified and soil parameters were determined with precision by performing several experiments in the laboratory. These parameters were needed as input parameters in the FEA simulations.

### Finite element analysis (FEA) using an advanced constitutive soil model

For the numerical simulation of the observed elephant footprint geometry (i.e., vertical displacements and diameter) FEA was used. In routine soil mechanics applications we normally derive settlements from the applied load. However, in the current study, we took the opposite approach by applying a specific type of so called back analysis (inverse analysis) in order to determine the load from the settlements. Inverse analysis is a well established tool in soil mechanics (for an overview see [37]). The FEA code used in this study considers three spatial dimensions and was originally developed for the analysis of deformations in geotechnical applications. Soil behavior is simulated in a non-linear elastic-plastic manner. Several soil models, e.g., the Mohr-Coulomb model and the hardening soil model [38], that differ in accuracy, are implemented in the FEA code to model the mechanical behavior of soil. The Mohr-Coulomb model is an elastic-plastic material model, that assumes a constant stiffness of the material (i.e., the stiffness of the soil) with the depth. However, this condition is generally not met by the mechanical behavior of soils. The Mohr-Coulomb model is mostly used in initial approaches to numerical modeling of soil mechanical behavior only, but it is physically wrong for solving deformation problems as in this research.

A more realistic material model for the simulation of the behavior of different types of soil is the hardening soil model. When soil is subjected to primary loading, it shows an increase in stiffness with increasing stress and develops an irreversible plastic strain. In contrast to the Mohr-Coulomb model, the hardening soil model implements the stress dependent stiffness behavior of the soils, i.e., the hardening of the soil is taken into account. In addition to the material parameters used in the Mohr-Coulomb model, i.e., friction angle 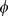 [°], cohesion *c* [kN/m^2^], dilatancy 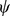 [°], the hardening soil model requires further input parameters. These include the stiffness modulus 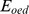 [kN/m^2^] for primary compression loading (derived from one-dimensional compression tests), the unloading and reloading stiffness modulus 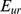 [kN/m^2^] (derived from one-dimensional compression tests), as well as the deviatoric stiffness 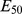 [kN/m^2^] (derived from triaxial tests). In reality, all loading conditions and loading directions may occur simultaneously, depending on the spatial position of an observation point. Therefore a constitutive model as used in this study is required that automatically analyzes the loading conditions and applies the relevant stiffness. Considering the fact that stiffnesses may vary by a factor of 7 to 10, we have to admit that less realistic soil models than the hardening soil model cannot be used for quantitative analyses. The required input parameters were determined in standard soil mechanics laboratory experiments that we performed with the material used as subsoil in the elephant field experiment.

### Method of digital image correlation (DIC)

**Figure 1:**
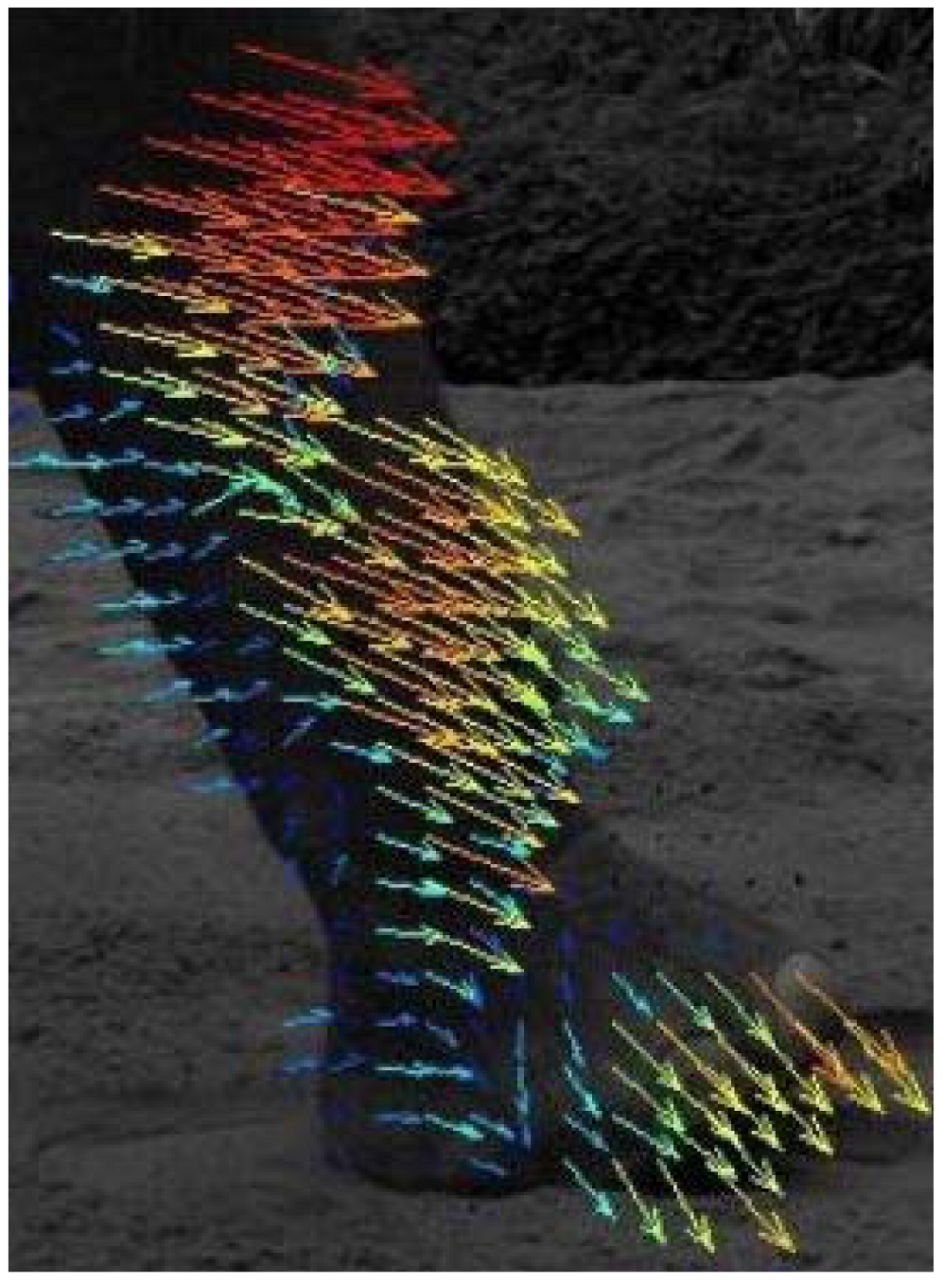
Vectors of displacement of elephant's forelimb obtained by DIC technique. The vectors illustrate the amount (length and color of arrows) and direction (orientation of arrows) of displacement.

As noted, the stress transmitted to the subsoil during animal walking has a dynamic and a static component. Subsoil deformation is a consequence of the maximum load, which either corresponds to the maximum static load 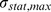 or to the sum of dynamic load and the corresponding static load 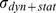. To determine the velocity of the elephant's foot at the time of contact with the subsurface, the DIC technique was used. The elephant's walk was recorded by a high speed camera (Casio Exilim EX-F1, 60 frames per second) and deformation of pixel clusters was analyzed for the defined time interval (Figure 1). See reference [39], for details of the DIC technique. The velocity vectors obtained by the DIC technique permit calculation of the dynamic stress applied to the subsoil based on the following equation:
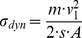
(1)where *m* [kg] is the mass in motion (i.e., the weight distributed over the limb considered); 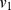 [m/s] is the velocity of the mass (i.e., the velocity of the limb) on impact on the subsoil; 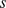 [m] is the path of deceleration (i.e., the deformation of the subsoil); and 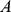 [m^2^] is the area of the foot obtained from footprint geometry. If the state of dynamic loading corresponds to the maximum load, a factor 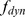 [–] can be obtained that relates 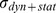 to 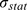: 
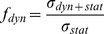
(2)


Thus, the stresses determined by FEA (i.e., 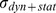) can then be related to the weight of the elephant: 
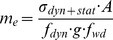
(3)


where 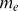 [kg] is the mass of the elephant; 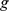 [m/s^2^] is the acceleration of gravity; and 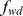 [–] is the factor considering weight distribution on the limbs, i.e., gait, by relating the mass carried by the particular limb (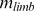 [kg]) to the total mass (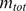 [kg]): 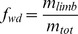
(4)


In summary, the factors 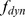 [–] and 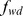 [–] differ for varying loading situations (i.e., combination of footfalls and walking velocity), but do not depend on the total mass of the elephant. Thus, application of Equation 3 to weight estimation of any other animal requires considerations of the anatomical characteristics and locomotion patterns of the trackmaker.

### 3D scanner

**Figure 2:**
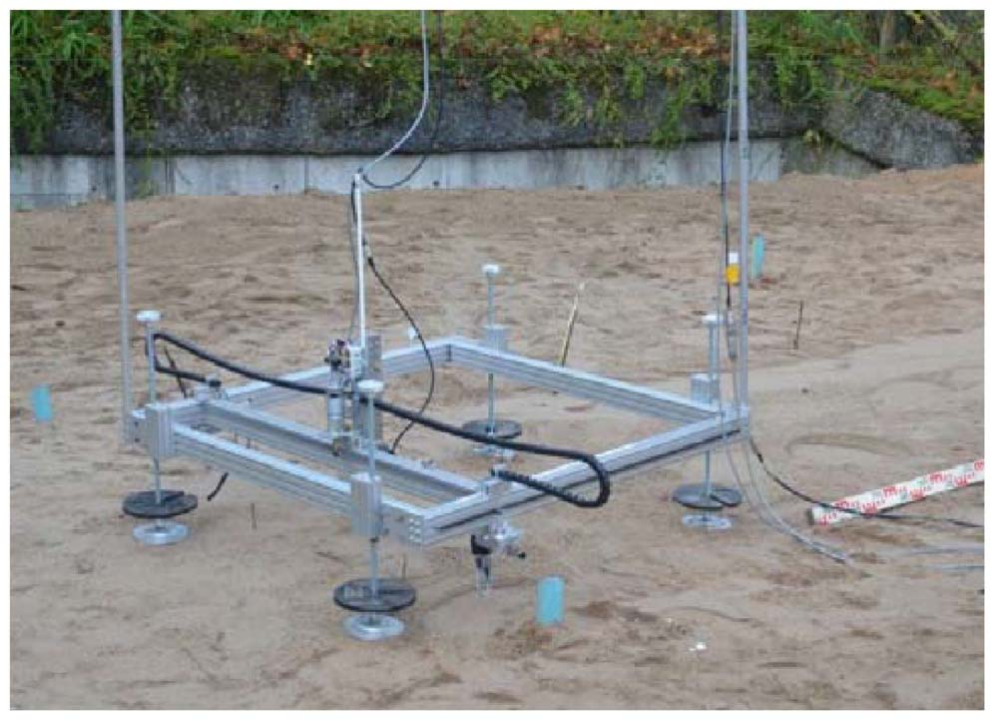
3D laser scanner developed and custom-built for recording animal tracks. The scanner covers an area of 800×800 mm.

Footprint geometry was captured with a portable laser scanner designed and constructed for this purpose. The scanner (see Figure 2) covers an area of 800×800 mm. The 3D surface scan provides very precise (± 75 *µ*m) information of the settlements in the subsoil produced by the weight of the elephant. This information is later needed for calculating the weight of the elephant using FEA.

### Classification of the soil used and derivation of soil parameters

**Figure 3:**
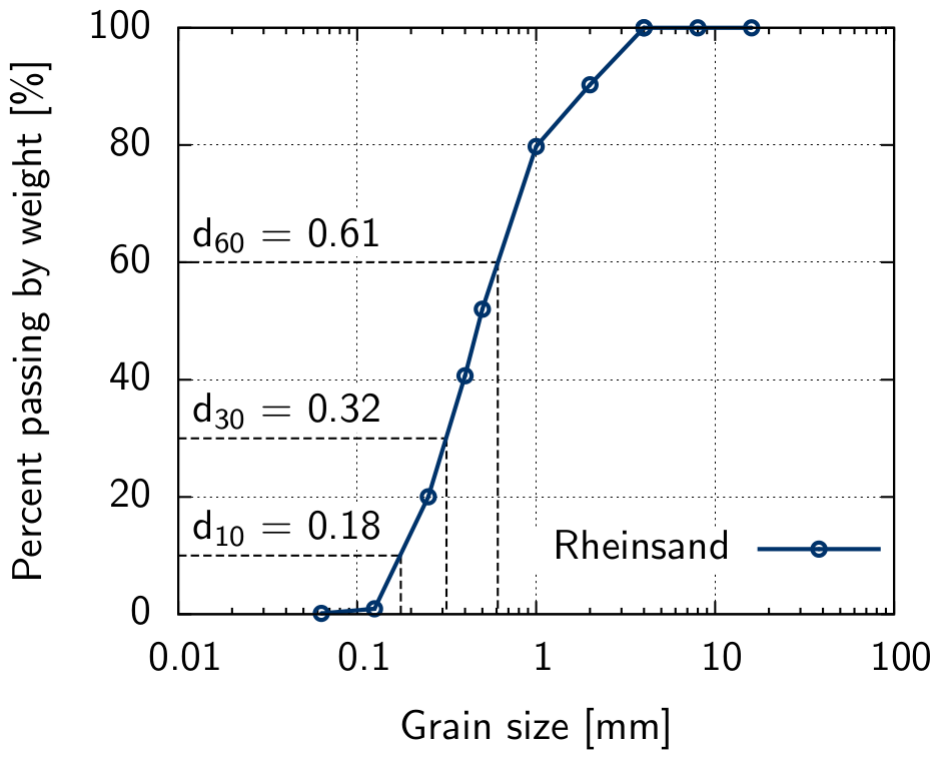
Grain-size distribution of Rhine sand. Grain sizes are given for characteristic values, i.e., for 10% (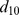), 30% (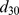), and 60% (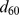) of the sand passing the corresponding mesh size by weight.

It is important to note that the general approach (including its accuracy) suggested in this paper does not depend on the type of subsoil. Different constitutive models are available and well validated in soil mechanics to consider, for example, cohesive soils or low permeability soils including consolidation analysis [40]. The sediment used in the neoichnological experiment was the so called Rhine sand. The grain-size distribution of Rhine sand is given in Figure 3. As can be seen from the grain-size distribution curve, grain-sizes range between 0.1 and 4.0 mm in diameter. The estimated coefficient of curvature 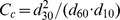 and the coefficient of uniformity 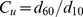, lead to the conclusion that the sediment is a poorly graded medium sand. Based on Hazen's formula[41], a permeability coefficient of *k* = 0.0003 m/s was calculated. The loose density was found to be 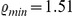 g/cm^3^, and the dense density was found to be 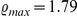 g/cm^3^, which correspond to a loose void ratio of 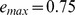 and a dense void ratio of 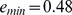.

Several tests are available in soil mechanics to measure the stress-strain behavior of a soil, e.g., the isotropic compression test, the one-dimensional compression test, the triaxial test, and the direct shear test [42].

**Figure 4:**
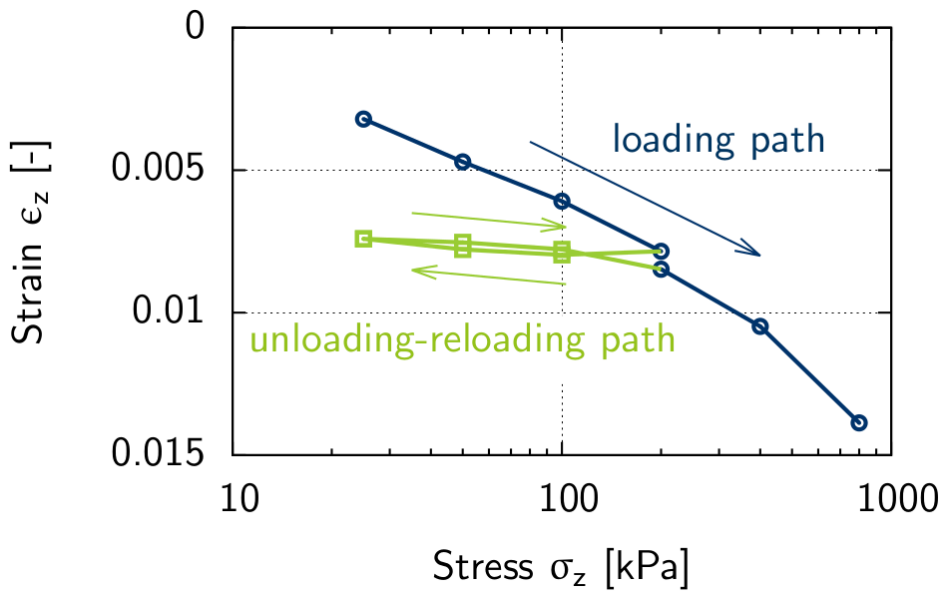
One dimensional compression and rebound test results for Rhine sand with an initial density of e = 0.6. Initial loading was conducted towards a value of 200 kPa followed by an unloading-reloading path down to 25 kPa. Initial loading was then continued towards a value of 800 kPa.

**Figure 5:**
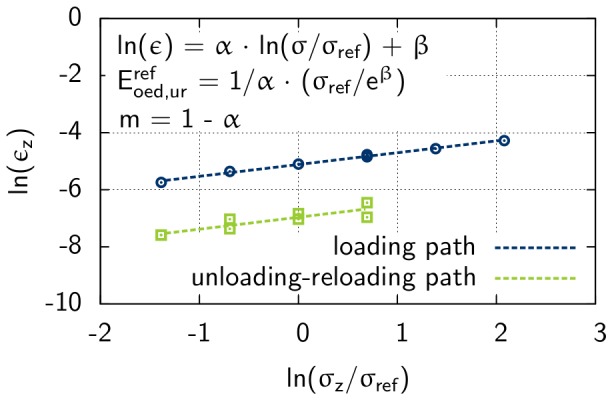
One dimensional compression and rebound regression analysis for Rhine sand with an initial density of e = 0.6. Parameters 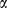 and 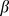 of linear functions for initial loading and unloading-reloading path lead to the stiffness value 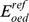 and 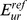, respectively.

In the present study, the stress-strain behavior of the soil was investigated using a one-dimensional compression and rebound test. This type of test is performed in conventional oedometer cells. Results derived from the one-dimensional compression and rebound test conducted on Rhine sand are shown in Figure 4 and Figure 5. This test includes the application of stress to a soil sample along the vertical axis, while the strain in the horizontal direction is restricted. To determine stress-strain behavior, the one-dimensional compression and rebound test is often used because it is simple to perform. We also used this test because the strain condition in the soil sample is approximately similar to the situation in the center of the load generated by the elephant's foot on the subsoil. Important parameters derived from one-dimensional compression test are the stiffness moduli 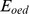 [kN/m^2^] and 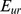 [kN/m^2^] that describe the stress dependent stiffness in a soil [43]. The stress dependent stiffness moduli 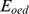 and 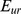 can be calculated based on Equation 5, where 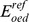 is the reference stiffness modulus for initial loading and 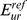 is the reference stiffness modulus for the unloading/reloading path determined for a reference stress 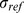 = 100 kN/m^2^ and *m* is a dimensionless parameter [44], [45]:
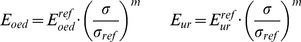
(5)


The parameter *m* and the normalized stiffness modulus 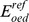 and 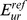 are derived from a regression analysis, that is presented in the diagram in Figure 5. To linearize the function of vertical net stress against strain 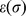, the logarithm of the strain 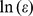 and the logarithm of the normalized stress 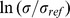 is used:
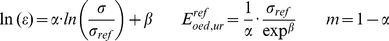
(6)


where 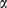 and 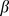 are the slope and the intersection with the y-axis, respectively.

**Figure 6:**
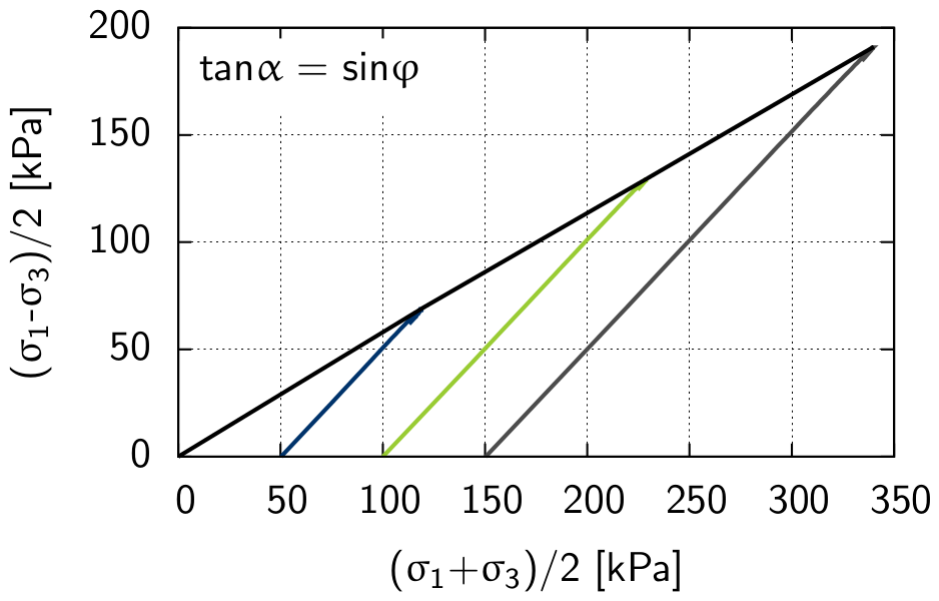
Triaxial test results for the determination of shear parameters of Rhine sand with an initial density of e = 0.6. Black line: Maximum shear stress is plotted against effective normal stress associated with cohesion c [kN/m^2^] and friction angle 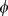 [°]. Blue, green and grey line: Stress paths for experiments conducted at 50 kN/m^2^, 100 kN/m^2^, and 150 kN/m^2^ confining pressure, respectively.

**Figure 7:**
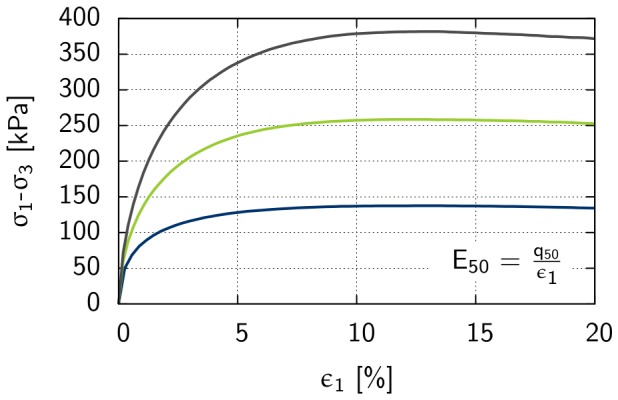
Triaxial test results for the determination of stiffness E_50_ [kN/m^2^] of Rhine sand with an initial density of e = 0.6. Blue, green and grey line: Deviatoric stress is plotted against axial strain for experiments conducted at 50 kN/m^2^, 100 kN/m^2^, and 150 kN/m^2^ confining pressure, respectively. The stiffness E_50_ is the secant stiffness over the first 50% of the deviatoric stress.

A triaxial test was performed to predict shear parameters such as friction angle, cohesion and angle of dilatancy [46]. Triaxial tests are conducted in a cell, where a cylindrical sample is subjected to a confining pressure 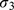 (radial stress). Increasing axial stress 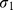 is applied to the sample by a vertical loading that causes shear failure in the sample. Figures 6 and 7 show results derived from triaxial tests conducted on Rhine sand at a cell pressure of 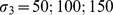 kN/m^2^ (i.e., the confining pressure), where maximum shear stress is plotted against effective normal stress (Figure 6), and deviatoric stress is plotted against axial strain (Figure 7). Based on Equation 7, the initial loading of the soil was described by the stress-dependent secant stiffness 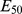 [kN/m^2^] (see Figure 7), that is the secant stiffness over the first 50% of the deviatoric stress:
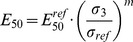
(7)where 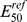 is the stress-dependent secant stiffness at reference stress 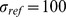 kN/m^2^. The friction angle was calculated from the maximum shear stress-effective normal stress diagram (see Figure 6) between the x-axis and the linear function through the points of maximum shear stress. The linear function intersects with the point of origin and leads to a cohesion value *c* = 0 kN/m^2^.

The hardening soil model parameters determined from triaxial and oedometer tests for Rhine sand with an initial density *e* = 0.6 (average density of Rhine sand in the field) are summarized in Table 1. For this type of subsoil material, i.e. sand, water content is of no significance, because additional strength and stiffness from capillary pressure is in the range of a few kN/m^2^ only. Also, permeability of the sand is so high that undrained conditions during loading do not have to be considered.

**Table 1:** Hardening soil model parameters.

Parameter	Rhine sand
*m* [–]	0.4
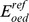 [MN/m^2^]	42
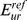 [MN/m^2^]	208
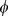 [°]	35
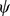 [°]	5
*c* [kN/m^2^]	0
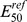 [MN/m^2^]	42

### Field experiment

The field experiment was carried out in the Zoological Gardens Wuppertal, Germany, with the tame African elephant cow Sweeny walking on a sand bed prepared in advance.

Because our goal was to back calculate the elephant's weight from a single footprint, some considerations on the gaits of elephants are in order here. Elephants differ remarkably from large hooved mammals in their locomotor repertoire by being confined to symmetrical gaits. In view of their great size (up to 5.5 tons), it is not clear whether this confinement depends on their unique size and thus is relevant for sauropods, or on some other reason. A simple theoretical consideration (detailed e.g. in [47]) may help. The speed reached in any gait is defined by the distance covered in one step cycle ('stride length') multiplied by cycle frequency. Since limb length as well as excursion angles are limited, great step lengths can only be reached by intercalating phases of suspension without ground contact into each step cycle. In combination with step frequency, this leads to a shortening of the ground contacts. Because the sum of impulses exchanged between the animal and the ground must be equal to its constantly acting body weight, the immediate consequence of a suspension phase are increased ground reaction forces. To avoid exceeding the strength limits of the limbs, suspension phases must be kept short or eliminated completely. In reference [48] the authors have calculated the ground reaction forces in dependence of the intervals available for ground contacts. According to these calculations, the mass of large sauropods alone compelled them to have used elastic damping mechanisms in order to avoid dangerous stressing of limbs even during a walk. This would have excluded the option of a further shortening of ground contact intervals which are typical for asymmetric gaits.

The gaits used by elephants for slow locomotion is a walk, the walk being a 4-beat rhythm with intervals between footfalls of 25% of cycle duration. To move faster, elephants change to a gait very similar to an 'amble' (a 4-beat rhythm with higher frequency than the walk) by elongating their steps [48], [49]. This is possible by intercalating a phase without ground contact, first with the hindlimbs and then with the forelimbs. This step elongation seems to be facilitated by marked elastic up and down-movements of the heavy head [48].

**Figure 8:**
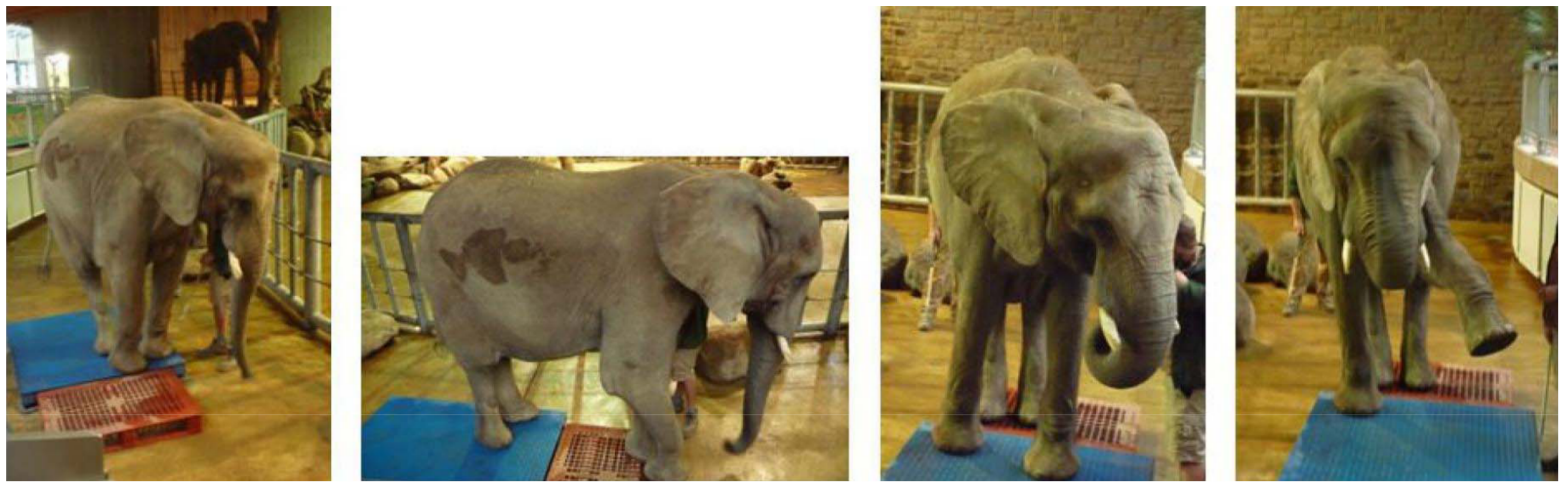
Weighing the elephant cow Sweeny. The following loads were measured: a) the elephant was standing with all limbs on the scale (m = 2530 kg), b) the load carried by both hindlimbs (m = 1125 kg), c) the load carried by both forelimbs (m = 1530 kg), and d) the load carried by one forelimb (m = 1390 kg).

Before the experiment the weight of Sweeny was carefully measured using the special scale kept in the elephant enclosure for this purpose. As can be seen in Figure 8, the weight was measured under several conditions to determine the weight borne by each limb of the elephant. The following loads were measured: a) the elephant was standing with all limbs on the scale (*m* = 2530 kg), b) the load carried by both hindlimbs (*m* = 1125 kg), c) the load carried by both forelimbs (*m* = 1530 kg), and d) the load carried by one forelimb (*m* = 1390 kg). If it is known from biomechanical considerations how the weight of the moving trackmaker is distributed on its limbs and which type of gait was used during track formation (according to 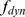 and 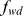 in Equation 3), analysis of just one print will be sufficient for determining the trackmakers weight.

**Figure 9:**
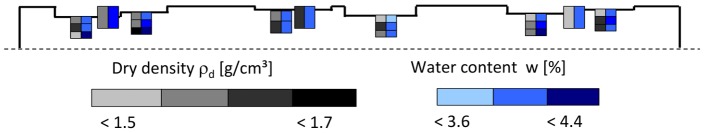
Results of dry density and water content profile measurements. Soil samples were obtained from the prepared test field by manual sampling with a metal tube. Samples were taken inside and outside several footprints, indicated by differing sampling depths, i.e., differing starting points of the top of the tube. Footprints are displayed schematically, for detailed information see Figure 11.

Prior to the experiment, a test field had been prepared for the elephant to cross. This consisted of an excavation in the elephant enclosure of 5.25 m in length, 2.20 m in width, and 0.90 m in depth, which was refilled with the experimental subsoil. The sand fill was prepared in three layers with each layer being compacted with a hand-pulled roller after dumping into the test field. Soil samples were obtained from the prepared test field by manual sampling with a metal tube and taken to the lab to determine density and water content. Dry density and water content of the samples are given in Figure 9. The average dry density was found to be 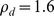 g/cm^3^. Homogeneity was an important experimental condition for the volume of soil influenced by the loading. This volume can be estimated as a cube with a side length of about twice the relevant loading dimension, which was foot diameter in our case. As noted, the subsoil was put into place in three layers, and each of these layers was verified for the target void ratio.

**Figure 10:**
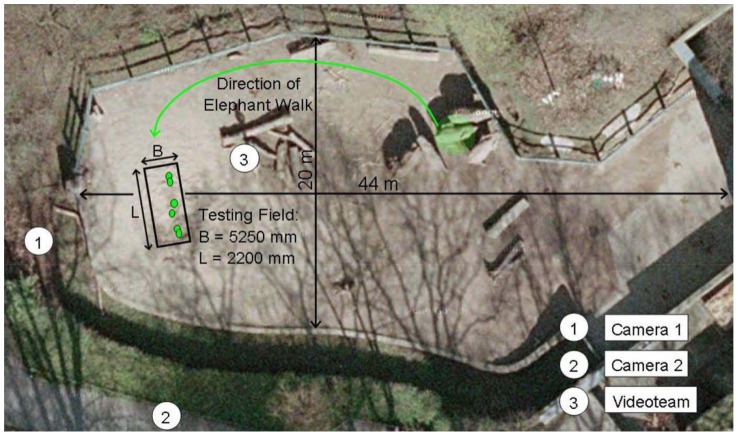
Satellite image of elephant enclosure (and elephants) at the Zoological Gardens Wuppertal including the testing field (www.google.de). Positions of the scanned footprints are marked in green within the prepared testing field.

**Figure 11:**
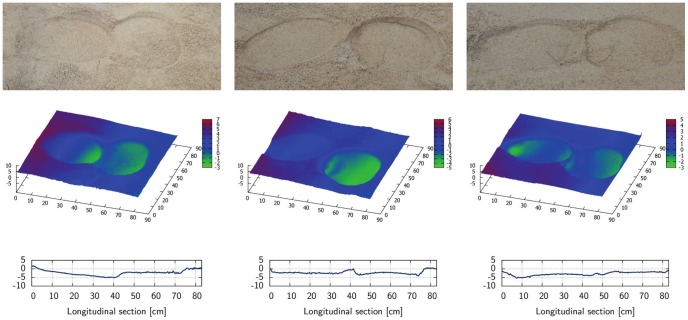
Capture of elephant footprints geometry using 3D laser scanner. A total of six footprints were scanned, i.e., three pairs, each of them consisting of one forefoot imprint (right) and one hindfoot imprint (left). Each pair is pictured by a photograph (top), 3D surface plot (center), and a 2D longitudinal section plot (bottom).

The elephant enclosure and the location of the test field is shown in Figure 10. Guided by one of her keepers, Sweeny walked across the test field during the experiment and left several footprints in the sand bed. A total of six footprints were scanned using the 3D laser scanner (see Figure 11). The area of the forefeet and hindfeet is about the same, whereas lengths ratio of forefeet to hindfeet is about 0.85, and the widths ratio is about 1.18. Visual analysis of the actual footprints and of the scanned prints indicates that the loading area is the same as the area imprinted on the subsoil. However, for practical reasons, we restricted the FEA to the footprints of the forelimbs. Based on the 3D scanner results, average footprint length is 0.32 m, average width is 0.30 m, and the average depths of the three scanned forefoot impressions is 0.020 m, 0.021 m, and 0.026 m, respectively.

## Results

**Figure 12:**
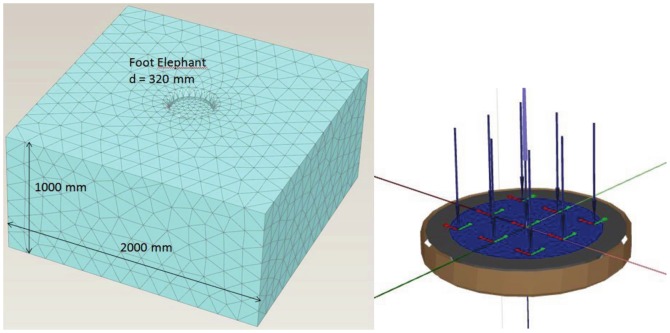
Geometry and generated mesh of the FEA model and interfaces. See text for a detailed description of the model.

Our 3D FEA model consists of a soil volume 2 m in width, 2 m in length and 1 m in depth and a circular plate 0.32 m in diameter that simulates the elephant's forefoot. Since the rigid plate differs from the soft sole of the elephant's foot, the numerical results for the vertical deformation were multiplied by a factor of 1/0.75 based on the DIN 4019-1 standard to take into account the flexible loading characteristics produced by the foot [50]. The geometry of the FE model, including the mesh generated, is given in Figure 12. The boundary conditions were set to the bottom of the model volume being fully fixed. The sides of the model were vertically unconstrained but fixed in all other directions. To simulate the subsoil-foot interaction, interfaces were introduced into the model around the circular plate. The outer interface were assigned the normal parameters of the subsoil, but reduced soil parameters were assigned to the inner interface to model smooth contact between the subsoil and the elephant's foot. The numerical simulation is a forward simulation, i.e., stress is applied through the plate to the soil, and then the settlements are derived. As described above, the hardening soil model was used for describing the mechanical behavior of the soil. The model input parameters were experimentally determined as described above.

**Figure 13:**
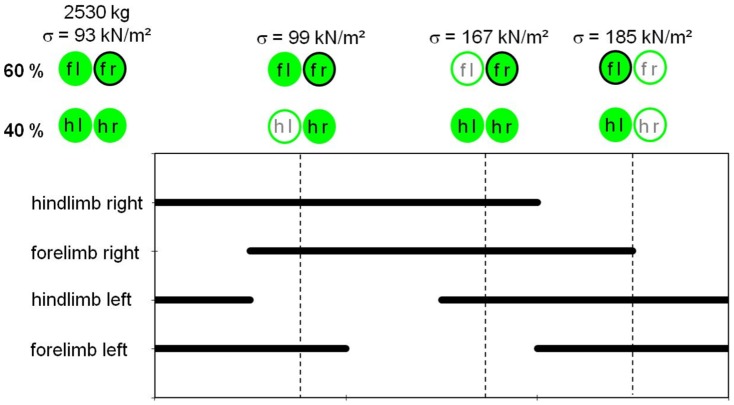
Sequence of footfalls in elephant walk after [5]. The static loading conditions (loading steps 1 to 4) simulated by FEA are marked and quantified within the sequence. The leftmost loading step is loading step 1, with the elephant at a standstill. Black bars indicate ground contact of the respective foot. fl  =  left forefoot, fr =  right forefoot, hl  =  left hindfoot, hr  =  right hindfoot. See text for a detailed description of the loading steps.

Two approaches were used in the numerical simulations. The first approach included the numerical simulation of the vertical displacements of the subsoil by the elephant's weight. The calculation is based on the results of the gait analysis, the application of the DIC technique, and the elephant's weight. The numerical simulation was performed using several phases. The initial phase included the generation of initial conditions in the soil, i.e., the configuration of the initial geometry and the initial stress state (e.g., effective stresses, state parameters). In the second phase, the circular plate was activated, without applying stress to the soil. In the following phases, the stresses induced by the weight of the elephant were applied successively. From the sequence of footfalls in the elephant walk (see Figure 13), four scenarios of static loading were simulated as loads applied to the circular plate simulating the elephant's forefoot. Application of a stress of 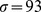 kN/m^2^ (loading step 1) simulated the standing elephant (i.e., the weight is distributed to all four limbs, where 60% of the weight is carried by the forelimbs and 40% is carried by the hindlimbs). Loading step 2 (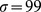 kN/m^2^) simulated the load on one forelimb with both forelimbs touching the ground but one hindlimb not touching the ground. Loading step 3 (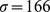 kN/m^2^) simulated the load on one forelimb with the other not touching the ground but both hindlimbs touching the ground. Loading step 4, representing the maximum static stress 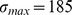 kN/m^2^ below the forefoot, simulated only one forelimb and one hindlimb touching the ground, as when the animal was progressing in a walk. In a final step (loading step 5), we added the dynamic component of the foot to the model by introducing the relevant stress 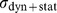 for the simulation of the settlements, i.e., the sum of the static stress of loading step 2 and the dynamic stress:
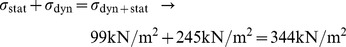
(8)The factors 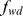 and 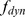, which determine the stresses applied during the loading steps according to Equation 3 are summarized in Table 2.

**Table 2:** Factors 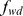 and 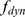 determining total mass distribution on the limbs during the elephant's walk.

	Forelimb	Hindlimb
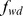		
4 limbs	0.3	0.2
3 limbs (2 fore-, 1 hind-)	0.32	0.36
3 limbs (1 fore-, 2 hind-)	0.54	0.23
2 limbs (1 fore-, 1 hind-)	0.6	0.4
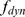	3.5	1.6

**Figure 14:**
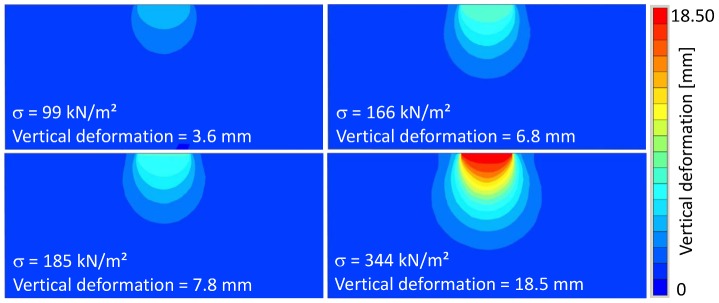
Vertical sections of FEA model at loading steps 2 to 5. Colors indicate amount of deformation.

**Figure 15:**
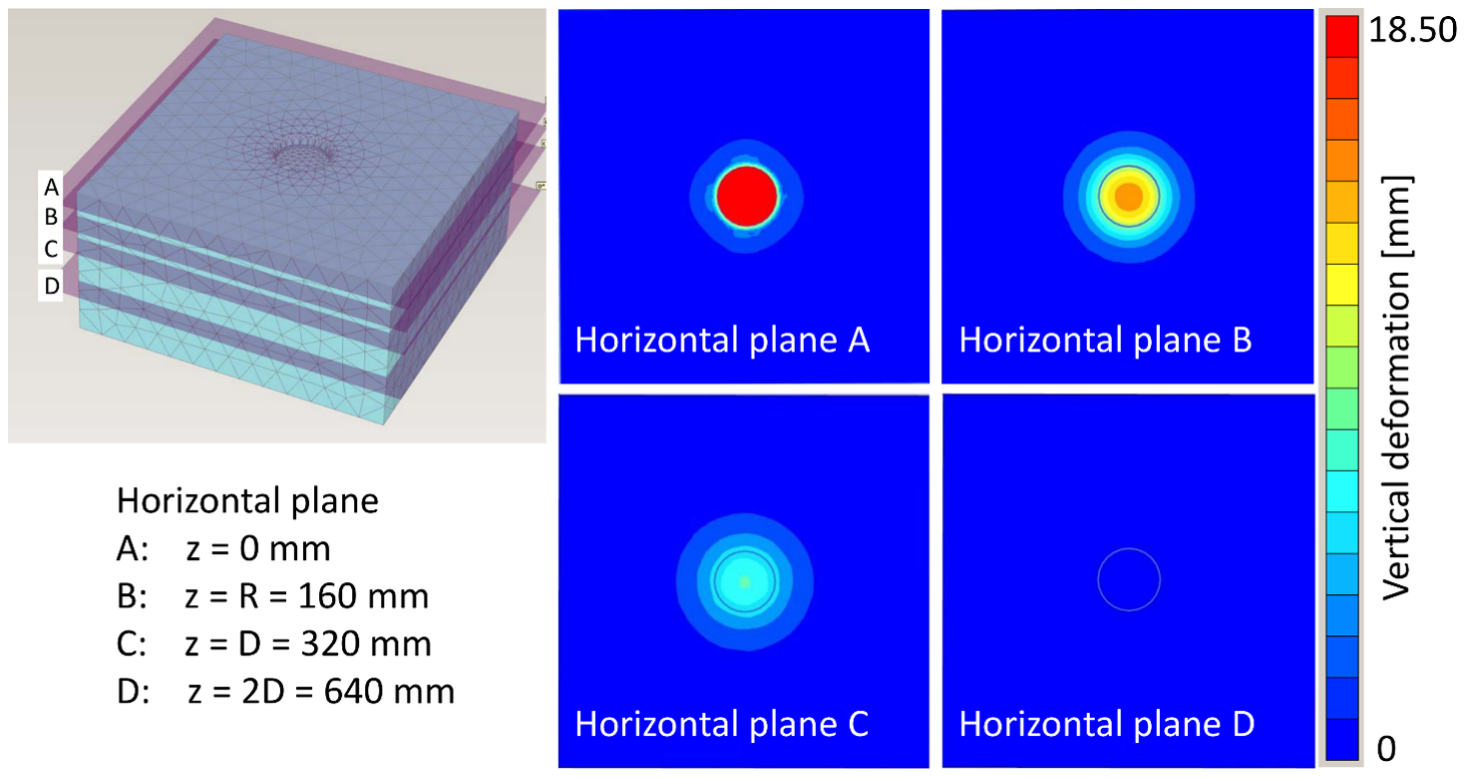
Four horizontal sections of FEA model of loading step 5. Horizontal plane A is at surface, horizontal plane B is at the depth of the radius R of the circular plate that was loaded to simulate the elephant's foot, horizontal plane C is at the depth of the diameter D of the circular plate, and horizontal plane D is at twice the depth of the diameter D of the circular plate. Colors indicate amount of deformation.

The results of the numerical simulation are shown in Figures 14 and 15, in which the vertical deformations are presented. For loading step 1, a deformation *u* = 0.003 m was calculated, loading step 2 resulted in a deformation of *u* = 0.004 m, loading step 3 in a deformation of *u* = 0.007 m, and loading step 4 in a deformation of *u* = 0.008 m. As expected the largest deformation was found for loading step 5 with *u* = 0.018 m.

**Figure 16:**
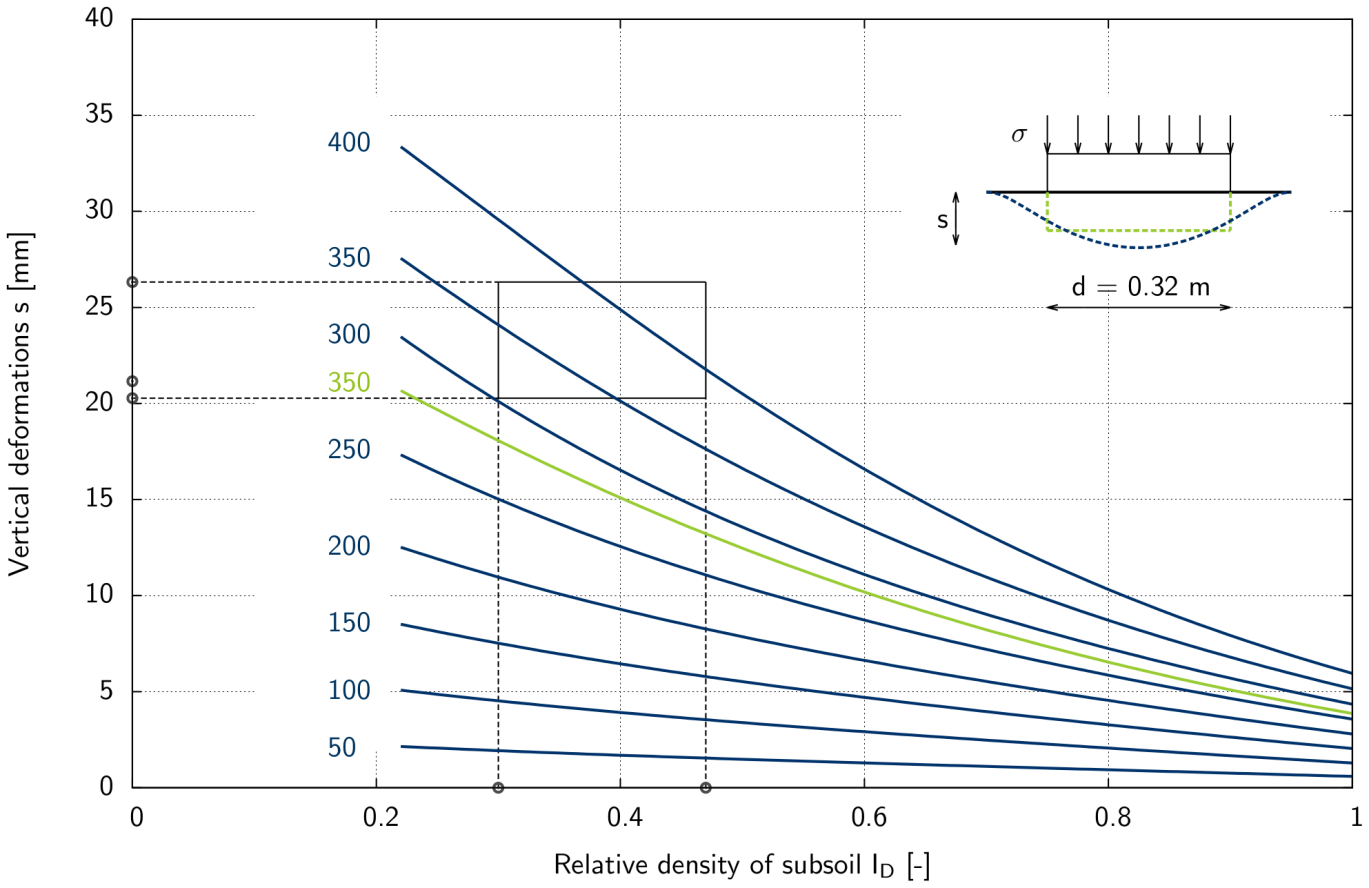
2D-plot of relative density versus settlements for back analysis of applied stress 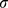 [kN/m^2^] by FEA for a circular plate (d = 0.32 m). The diagram applies to subsoil conditions of Rhine sand. According to the deformation characteristics illustrated at the top right corner of the diagram, blue curves apply to the flexible loading characteristics of the elephant's foot, and the green curve (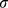 = 350 kN/m^2^ 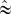 loading step 5) applies to rigid loading characteristics used in the FEA model. The relationship is detailed in the text. The range of stresses that can be back-calculated from in situ conditions of relative density of subsoil 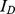 (0.3 and 0.47) and measured values of *s* (20.28 mm, 21.16 mm, and 26.32 mm) is marked by a box.

**Figure 17:**
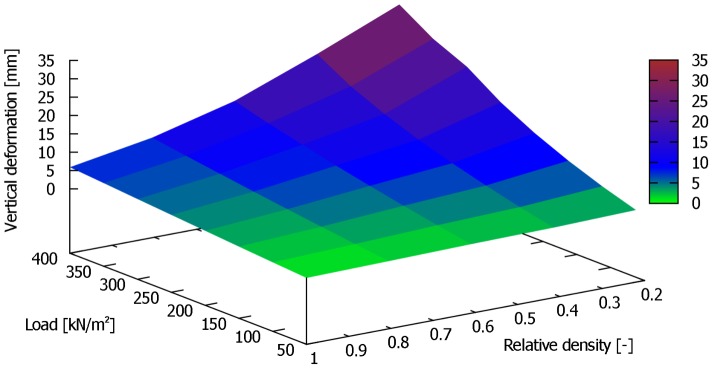
3D-plot of relative density versus settlements for back analysis of applied stress 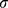 [kN/m^2^] by FEA for a circular plate (d = 0.32 m). The diagram applies to subsoil conditions of Rhine sand. This diagram can be used to estimate the load having produced a fossil footprint if the original subsoil parameters were the same as our experimental subsoil, Rhine sand.

In order to determine the weight of a dinosaur based on back analysis of vertical settlements, a second approach was developed. In this approach, numerical simulations were carried out for Rhine sand subsoil with relative densities of 
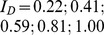
 and applied stresses of 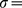 50; 100; 150; 200; 250; 300; 350; 400 kN/m^2^, respectively. The relative density is calculated as follows:
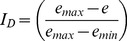
(9)where 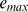 and 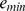 are the maximum and minimum void ratio of the soil and e is the void ratio of the soil. For each simulation, hardening soil model parameters were calculated from experimental results carried out on Rhine sand samples with the appropriate void ratio. In Figures 16 and 17, the results of the second approach are presented that allows determination of the stress applied to a specific subsoil and thus the total mass of an animal (see Equation 3). To use the diagram, only two values have to be known: the relative density of the subsoil 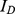 [–] and footprint geometry (i.e., vertical displacement and diameter). In the case of the elephant's footprints, the relative density of the subsoil was found to be between 0.30 and 0.47, and measured vertical displacements were between 0.020 m and 0.026 m. Using these results as input values in the diagram in Figure 16, applied stress with an average value of about 360 kN/m^2^ can be obtained. Using Equation 3, an average mass of about 2635 kg can be back-calculated from the geometry of the elephant footprints and the relative density of the soil.

## Discussion

The present study illustrates the successful application of soil mechanical concepts to the quantitative interpretation of the soil deformation represented by footprints. Two aspects have to be taken into account accurately: (1) the simulation of the behavior of the subsoil using corresponding soil parameters and (2) the relationship between applied stress and total mass of the animal. The constitutive soil model used in this study for FEA describes soil behavior in a most realistic manner since it takes into account stress and loading direction dependent soil stiffness. The geometry, initial conditions and boundary conditions of the model, as well as the input parameters characterizing soil behavior, influence the results of subsoil deformation and have to be accurately identified.

The present research study indicates that the dynamic component of the trackmaker has a significant influence on subsoil deformation. A factor of approximately 3.5 relating 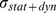 to 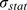 was identified using the DIC technique to quantify the velocity of the elephant's foot when coming into contact with the subsoil. The outcome of our numerical simulation is that the average vertical displacement 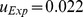 m measured in the field experiment is in good agreement with the numerically calculated vertical displacement 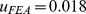 m as a result of the maximum applied stress 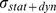.

## Conclusions

We conclude that a reliable method for weight reconstruction from footprints has been developed, implemented and validated. Our inverse approach, as shown in Figure 16 and 17, allows the stress applied to a specific subsoil to be determined. In addition, the total weight of an animal (see Equation 3) can be determined with an error of about 15%.

Our work represents a first step in the direction of back calculating the weight of extinct animals such as sauropod dinosaurs from their footprint. However, several additional footprint and subsoil characteristics have to be considered before reliable results can be obtained for fossils. These include geological processes that alter the original subsoil deformation such as the (1) influence of overburden pressure on subsoil deformations after the footprint was created, (2) identification of the type of fossil footprint (i.e., undertrack, overtrack, true track), (3) surface weathering, and (4) the soil profile, including constitutive parameters and layering of the subsoil. Accordingly, in ongoing research using micro-CT analysis, realistic stiffness parameters of fossil subsoils are estimated from the granulometric properties of the rock in which the footprint is preserved. It thus is clear that detailed sedimentological study must precede the soil mechanical approach in the study of sauropod footprints.
